# Comparative effectiveness of Propolis with chlorhexidine mouthwash on gingivitis – a randomized controlled clinical study

**DOI:** 10.1186/s12906-024-04456-8

**Published:** 2024-04-06

**Authors:** Shilpa Gunjal, Deepak Gowda Sadashivappa Pateel

**Affiliations:** 1 Division of Clinical Oral Health Sciences, School of Dentistry, IMU University, Bukit Jalil, Kuala Lumpur, 57000 Malaysia; 2 Department of Oral Pathology Oral Medicine, Faculty of Dentistry, MAHSA University, Bandar Saujana Putra, Jenjarom, Selangor 42610 Malaysia

**Keywords:** Propolis, Chlorhexidine, Mouthwash, Gingivitis, Plaque control

## Abstract

**Background:**

To assess and compare the effectiveness of propolis mouthwash with chlorhexidine mouthwash in the reduction of plaque and gingivitis.

**Methods:**

A single centre, latin-square cross-over, double masked, randomized controlled clinical trial was conducted on 45 chronic generalized gingivitis subjects who were chosen from the dental clinic of MAHSA University, Malaysia. A total of 45 subjects were randomly assigned into one of the three different groups (*n* = 15 each) using a computer-generated random allocation sequence: Group A Propolis mouthwash; Group B Chlorhexidine mouthwash; and Group C Placebo mouthwash. Supragingival plaque and gingival inflammation were assessed by full mouth Plaque index (PI) and gingival index (GI) at baseline and after 21 days. The study was divided into three phases, each phase lasted for 21 days separated by a washout period of 15 days in between them. Groups A, B and C were treated with 0.2% Propolis, Chlorhexidine, and Placebo mouthwash, respectively, in phase I. The study subjects were instructed to use the assigned mouthwash twice daily for 1 min for 21 days. On day 22^nd^, the subjects were recalled for measurement of PI and GI. After phase I, mouthwash was crossed over as dictated by the Latin square design in phase II and III.

**Results:**

At baseline, intergroup comparison revealed no statistically significant difference between Groups A, B and C (*p* > 0.05). On day 21, one-way ANOVA revealed statistically significant difference between the three groups for PI (*p* < 0.001) and GI (*p* < 0.001). Bonferroni post-hoc test showed statistically significant difference between Propolis and Chlorhexidine mouthwash (*P* < 0.001), with higher reduction in the mean plaque and gingival scores in propolis group compared to chlorhexidine and placebo groups.

**Conclusions:**

Propolis mouthwash demonstrated significant improvement in gingival health and plaque reduction. Thus, it could be used as an effective herbal mouthwash alternative to chlorhexidine mouthwash.

**Trial registration:**

The trial was retrospectively registered on 25/07/2019 at clinicaltrials.gov and its identifier is NCT04032548.

## Background

Gingivitis is widely prevalent among adults around the world [1]. According to National Oral Health Survey for Adults 2010, Malaysia, 94% prevalence of gingivitis and its sequelae in the adult population (CPITN ≥ 1) was observed, [2] while 2010 national USA survey reported 86% of adults in America with clinical attachment loss of ≥ 3 mm [3]. Gingivitis, primarily caused by the pathological changes induced by the dental plaque, is considered to be an early stage of periodontitis [4]. Uncontrolled bacterial activity associated with dental plaque accumulation on the teeth and gums usually progresses into periodontal disease and is one of the most common causes of tooth loss [5]. If left unattended, invariably leads to periodontitis. The factors which indicate this progression can range from the signs like bleeding during brushing halitosis and mobility while eating [6]. So, it is quite important to prevent periodontitis and to control gingivitis. Maintenance of oral hygiene plays an essential role in the prevention of the tooth loss due to periodontitis.

The traditional primary preventive measures widely followed utilization of mechanical aids like toothbrush and toothpaste and the existence of newer mechanical aids like powered flossing [7], end-tufted brushes [8], and oral irrigation devices [8, 9]. As an adjuvant to these mechanical methods, certain chemical antimicrobial solutions are used to maintain good oral hygiene status. Chlorhexidine mouthwash is the most common antibacterial mouthwash marketed worldwide and considered the gold standard of mouthwash formulations. However, negative side effects such as staining of teeth, taste alteration and oral mucosal irritation have been associated with prolonged chlorhexidine use [10]. Increased consideration has been given to alternative mouthwash formulations with natural ingredients, including various herbs. Triphala, piper betel, mouthwashes are herbal formulations which have shown good plaque-reducing efficacy comparable to chlorhexidine [11]. The reason why numerous mouthwashes are being tested and investigated is primarily to look for a superior alternative to chlorhexidine [12]. However, literature review reveals that the current data is still inconclusive and there is need for further evidence through high-quality research to quantify the applicability of herbal mouth wash in relation to chlorhexidine [13]. Propolis is a resinous honeybee product that is used by bees to repair defects in beehives. The medicinal properties of propolis have been known since 300BC and is used as folk medicine in Balkan countries as reported by Haydak in 1950 [14]. Propolis exhibits anti- inflammatory, antibacterial, antifungal and antiviral activities [15–17]. So, the application of propolis with respect to gingivitis and periodontitis are being investigated. Propolis toothpaste is effective in reducing gingival inflammation in patients with oral clefts and dental appliances [18]. Furthermore, on comparison of efficacy of propolis chewing gum and propolis mouthwash, mouthwash showed greater reduction in plaque and gingival indices scores [19]. Thus, propolis can be used as an effective alternative to chlorhexidine in patients with fixed appliances [20].

Numerous studies are carried out to know the role of propolis on oral health. However, due to the fact that the propolis from different regions of the world vary in chemical constituents due to differences in climate and local flora, [21] which justifies further need of studies in different parts of the world. Malaysian propolis is found to possess anti-oxidative and broad-spectrum antibacterial effects that inhibits growth of bacteria in vitro [22]. Limited studies have quantified differences between Malaysian propolis with other propolis variants with respect to oral hygiene. In-vitro studies have shown variations in the antibacterial, antifungal, and antioxidant properties of different types of propolis [23]. Exploration of literature revealed no research has evaluated the effect of Malaysian propolis as an antibacterial mouthwash on gingivitis. Thus, the present study was designed to evaluate the effectiveness of Malaysian Propolis as a mouthwash in the reduction of plaque and gingivitis.

- Research hypothesis: Propolis mouthwash is effective in reducing gingival and plaque scores in gingivitis.
- Research question: Is propolis mouthwash effective in reducing plaque and gingival scores in gingivitis?

## Methods

### Ethical aspects

The study was done in accordance with the Declaration of Helsinki and approved by Research Review and Ethics committee of MAHSA University, Malaysia (RMC/AL02/2017). The Consolidated Standards of Reporting Trials (CONSORT) checklist is available as supporting information. The trial was posted on 25/07/2019 at ClinicalTrials.gov and its identifier is NCT04032548. All participants gave written informed consent, and they were informed about the details of procedure, risks, and benefits of the study.

### Study design

**Fig. 1:**
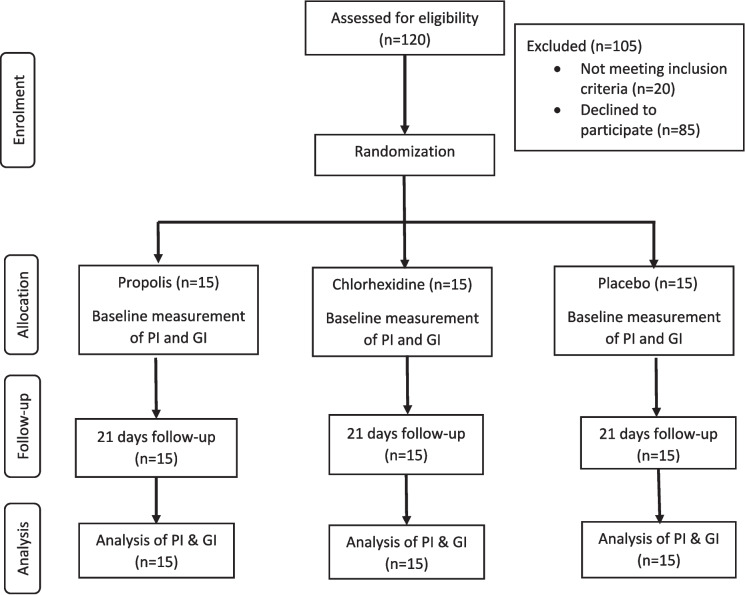
Flow chart of study participants in each phase

A single-centre, Latin-square cross-over, double masked (investigators and statisticians), randomized controlled clinical trial was conducted to assess the effectiveness of propolis mouthwash on chronic generalized gingivitis. In latin square cross-over design, each participant is treated as a block, which helps to control for individual differences that could impact the gingival and plaque scores of the study. The sequential order of the study protocol followed in the present study is shown in Fig. 1.

### Study population and selection criteria

Subjects were recruited between March 2019 to June 2019. The follow-up visit of the last subject was completed in December 2019. A total of 124 subjects were screened and assessed for eligibility (49 did not meet the inclusion criteria, 30 subjects declined to participate). Forty-five subjects (30 females and 15 males) who met the inclusion criteria, gave consent to participate were recruited from dental clinic of MAHSA University. Adults age range of 18–30 years were enrolled if they have the following inclusion criteria: (1) good systemic health, (2) Gingival index > 1 (3) Periodontal pocket depth ≤ 3 mm (4) Clinical attachment loss “0” (5) Provision of written informed consent. Exclusion criteria were: (1) Severe periodontal disease, as characterized by purulent exudates, generalized mobility, and/or severe recession (2) Any condition that requires antibiotic premedication for the administration of a dental prophylaxis (3) Self-reported pregnancy, intent to become pregnant during the study, or breast-feeding (4) Any diseases or condition that could be expected to interfere with the safe completion of the study (5) History of antibiotic use in the previous 3 months (6) Individuals with orthodontic appliances or prosthetic appliances that would interfere with evaluation 6) using tobacco products in any form (7) allergy to chlorhexidine or any of the components in the tested products, (8) systemic diseases (e.g., diabetes mellitus, hypertension and immunological disorders).

Subjects were instructed to avoid systemic antibiotic therapy, local antimicrobials and use of toothpastes containing antimicrobial agents. They were also advised to avoid alcohol and tobacco during treatment. Patients received detailed information on the instructions for plaque control including tooth brushing and flossing. All the subjects received the same brand of toothpaste (Colgate® Strong Teeth toothpaste, Malaysia) and toothbrush (Colgate sensitive soft bristle toothbrush, Malaysia) during the study period.

### Randomization and allocation concealment

**Fig. 2:**
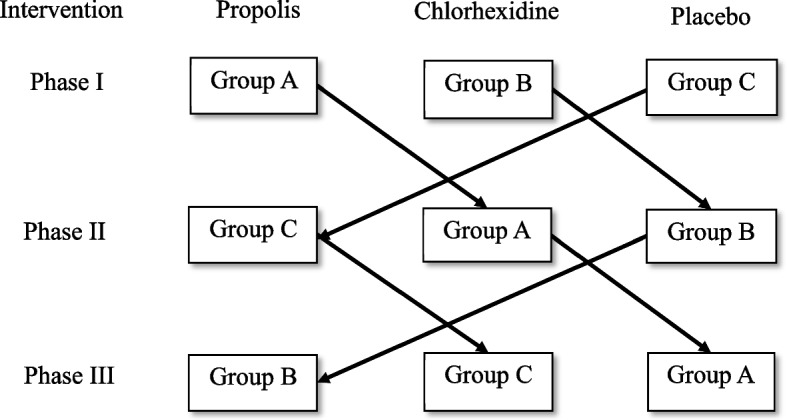
Latin square cross-over design depicting sequence of order for all the three groups

A total of 45 patients were randomly assigned into one of the three different groups (*n* = 15 each) using computer generated random allocation sequence (D.P): Group 1 PR mouthwash; Group 2 included CH mouthwash; and Group 3 included PL mouthwash. The random allocation was concealed by having a person not involved in the study. The clinician who performed all measurements was blinded to the treatment arms to the patients (S.G). The randomization codes were not broken until data had been collected. As the study design was a crossover utilizing Latin square design; each group was exposed to all three interventions in a phased manner (block randomization) as shown in Fig. 2. During phase I, three mouth rinses were randomly allocated to three different groups using the lottery method. Further, in phase II and phase III, the groups follow the sequence as shown in Fig. 2 as it is Latin square design.

### Rinse formulation

Rinse formulation: Propolis rinse 5% was prepared in the College of Pharmacy, MAHSA University. The formulation included 5% propolis, mint flavour, propylene glycol, sorbitol, and water. Propolis rinse was made by using propolis from Malaysia (NHF, Malaysia). The placebo rinse was prepared like propolis mouth rinse except the active ingredient propolis. Readily available Oradex antibacterial mouthwash [(0.12% chlorhexidine gluconate w/v), Fortune Laboratories Shd Bhd, Selangor, Malaysia] was used as the positive control.

### Training and calibration

Prior to the start of the study, five subjects were examined for GI and PI twice within 24-hour interval. The calibration was accepted if the measurements at baseline and the 24-hour interval were close to mean score of 0.5 at the 95% level.

### Clinical measurement

Supragingival plaque and gingival inflammation were assessed by full mouth Silness and Loe Plaque index (PI) [24] and Loe and Silness Gingival index (GI) [5]. Both the clinical parameters were measured at baseline and 3 weeks after intervention by one examiner who was calibrated for PI and GI by Kappa index which resulted in agreement of 0.85 and 0.87 respectively [25].

### Intervention protocol

The mouth rinses namely, 0.12% Chlorhexidine mouth rinse, Placebo mouth rinse (self-prepared), and 5% Propolis mouth rinse (self-prepared) constituted three interventions.

Subjects were instructed to rinse for 21 days [26], twice daily, morning after breakfast and night before going to bed, with 10 ml (undiluted) of the assigned mouth rinse for 1 min and then expectorate the rinse. A measuring cup was provided to all the subjects to dispense 10 ml of the assigned mouth rinse. All the subjects were instructed to avoid drinking or eating for minimum of half an hour after rinsing. On day 22, subjects were instructed to rinse once in the morning after breakfast. The use of mouth rinse was followed by a washout period of 15 days during which participants were asked to stop using the assigned mouth rinse. After the washout period of 15 days the selected subjects were assigned to the next mouth rinse in a phased manner (Fig. 2). During the entire course of the study, all the participants received Colgate toothbrush and Colgate total toothpaste and they were instructed to brush twice daily, once in the morning after breakfast and once at night after dinner.

### Compliance

All the participants included in the present study received a checklist to note and record the assigned mouth rinse for 21 days, along with timings (morning and night) to monitor compliance with the use of mouth rinse. The checklist also had an additional column to record any side effects experienced during the intervention period.

### Sample size calculation

Sample size calculation for randomized controlled trial was determined to be 15 in each group which would provide a power of 80% and level of significance at 5% with expected mean difference of 2.281 and standard deviation of 2.563. Based on the above calculation, the minimum sample of 12 is required in each group. Considering an attrition of 20%, 15 subjects in each group and a total of 45 subjects were recruited.

### Statistical analysis

The data was cleaned, coded, and analyzed using Statistical Package for Social Science (SPSS) version 27 (SPSS Inc., Chicago, IL, USA). The normality of data was checked using the Shapiro Wilk test with *p* > 0.05, indicating fulfilment of the normality assumption. Paired-t test was performed to assess the significant difference between baseline and after intervention for both plaque index and gingival index for PR, CH, and PL groups. Analysis of variance (ANOVA) was performed to compare the significant difference in the mean plaque and gingival sore between PR, CH, and PL groups. Post-hoc test was performed using the Bonferroni method to determine statistically significant difference between the two groups. *p* < 0.05 was considered for statistical significance.

## Results

In the latin square cross-over design, data was analysed by compiling the observations for each intervention at each phase, totalling 45 participants’ data for each group at baseline and post-intervention. Demographic characteristics of study subjects are presented in Table 1. A total of 45 subjects participated in the study of which 16 (35.55%) were males, and 29 (64.45%) were females. The mean ± SD age for all the study participants was found to be 23.867 ± 0.726 years.

**Table 1:** Demographic characteristic of study participants

Characteristics		
Age (years)	Mean	23.867
	Standard deviation	0.726
	Minimum	22
	Maximum	25
Gender n (%)	Male	16 (35.55%)
	Female	29 (64.45%)

### Gingival index

At baseline, the mean and standard deviation GI scores were 1.31 ± 0.24, 1.30 ± 0.25, and 1.24 ± 0.18 for PR, CH, and PL groups respectively and there was no statistically significant difference between the groups (*p* = 0.274). Paired-t test revealed statistically significant difference from baseline to after intervention in PR (*p* < 0.001), CH (*p* < 0.001) & PL (*p* = 0.038) for mean gingival index scores. (Table 2). One-way ANOVA revealed statistically significant difference between the three groups after intervention for the mean gingival index scores (*p* < 0.05) (Tale 2) followed by a post-hoc test by Bonferroni method which revealed statistically significant difference for all pair-wise comparison (*p* < 0.05) (Table 3). There is higher gingival score reduction in the PR group (mean difference of 0.66) when compared to CH (0.39) and PL group (0.09).

**Table 2:** Comparison of mean PI and GI at baseline and after 3 weeks for PR, CH and PL groups (within and between groups)

Variables	Timepoint (n)	Groups									One-way ANOVA (*p*-value)
		PR			CH			PL			
		Mean ± SD	Mean differeence 95%CI	*p*-value	Mean ± SD	Mean differeence 95%CI	*p*-value	Mean ± SD	Mean differeence 95%CI	*p*-value	
PI	Baseline (45)	1.37 ± 0.27	0.64 (0.537, 0.743)	0.001*^a^	1.35 ± 0.28	0.41 (0.303, 0.509)	0.001*^a^	1.30 ± 0.24	0.05 (0.003, 0.105)	0.038*^a^	0.414^b^
	3 weeks (45)	0.7 ± 3 0.25			0.90 ± 0.17			1.24 ± 0.24			0.001*^b^
GI	Baseline (45)	1.31 ± 0.24	0.66 (0.575, 0.748)	0.001*^a^	1.30 ± 0.25	0.39 (0.287, 0.500)	0.001*^a^	1.24 ± 0.18	0.09 (0.036, 0.146)	0.002*^a^	0.274^b^
	3 weeks (45)	0.65 ± 0.26			0.90 ± 0.20			1.15 ± 0.24			0.001*^b^

*PR *Propolis, *CH *Chlorhexidine, *PL *Placebo, *PI *Plaque index, *GI *Gingival index

*p < 0.05 statistically significant

^a^Paired t Test

^b^One-way ANOVA

**Table 3:** Pairwise comparison of mean PI and GI between PR, CH and PL groups after intervention

Groups	Mean difference (95% CI)	*p*-value
PI		
PR vs. CH	-0.22 (-0.331, -0.102)	< 0.001^*^
PR vs. PL	-0.51 (-0.628, -0.399)	< 0.001^*^
CH vs. PL	-0.25 (-0.411, -0.183)	< 0.001^*^
**GI**		
PR vs. CH	-0.25 (-0.373, -0.133)	< 0.001^*^
PR vs. PL	-0.50 (-0.618, -0.378)	< 0.001^*^
CH vs. PL	-0.25 (-0.365, -0.125)	< 0.001^*^

*PR *Propolis, *CH *Chlorhexidine, *PL *Placebo, *PI *Plaque index, *GI *Gingival index

*p < 0.05 statistically significant (post-hoc test applied using Bonferroni method)

### Plaque index

At baseline, the mean and standard deviation plaque scores were 1.37 ± 0.27, 1.35 ± 0.28, and 1.30 ± 0.24 for PR, CH, and PL groups respectively and there was no statistically significant difference between the groups for plaque scores (*p* = 0.414). Paired-t test revealed statistically significant difference from baseline to after intervention in PR (*p* < 0.001), CH (*p* < 0.001) & PL (*p* = 0.002) for the mean plaque index scores. (Table 2). One-way ANOVA revealed statistically significant difference between the three groups after intervention for mean plaque index scores (*p* < 0.001) (Table 2) followed by a post-hoc test by bonferroni method which revealed statistically significant difference for all pair-wise comparison (*p* < 0.001) (Table 3). There is higher plaque score reduction in the PR group (mean difference of 0.64) when compared to CH (0.41) and PL group (0.05).

## Discussion

The quest for an alternative natural product for a standard allopathic medicine is always there, especially to avoid the side effects associated with them. This is applicable to the field of oral health as well. Propolis is one such natural product that has gained a lot of attention and has been extensively studied in the fields of medicine and dentistry. Propolis is a natural honeycomb product, and a literature review indicates that propolis has already found its applications in most of the dental specialties like, the Periodontology and oral health [27], Oral Medicine [28], Oral surgery, [29] Orthodontics [30]. Endodontics [31]. Prosthodontics, [32] and restorative dentisty [33]. One may consider propolis to have a positive societal significance in helping to maintain oral health considering the facts that Malaysia is reported to have a high rate of periodontitis [34] and simultaneously the Malaysian bee sector is expanding [35]. So, it is interesting to study how propolis, with its potential antimicrobial properties, could be utilized in oral health care, especially in the context of maintenance of gingival health.

The biologic activity of the propolis is mostly associated with the flavonoids (flavonols, falavonones) phenolics, and aromatics present in the propolis [36]. However, the composition varies and is complex, and it is determined by the origin and type of bee that produced it as well as the collection season [28]. The composition of propolis from different regions of the world tends to have similarities in their basic constituents but shows variations [37]. The Malaysian propolis used in this study was sourced from a local commercial product containing 1.3% bee propolis and 1.0% *Apis cerana fabricius*. The sole known raw product was *Apis cerana fabricius*, Asian honeybees originating from East Malaysia, and the manufacturer did not provide the raw product’s processing specifications.

The present study implemented the Latin square design which is an extension of randomized complete block designthat can be used to control sources of extraneous variation or nuisance factors. By having each participant receive every treatment, intra-subject variability is reduced, which can result in greater statistical power to detect differences between treatments. The present study design is analogous to the equivalence clinical trial [38] which checks the applicability and clinical relevance of Malaysian Propolis as a mouth wash to reduce gingivitis and periodontitis in comparison with the standard CHX mouth wash.

The results of the present study revealed that gingival inflammation via plaque index (PI) reduced drastically and resulted in a higher improvement of gingival index (GI) in the propolis group compared to chlorhexidine, and both were significant compared to the control group (Table 2). The results of the present study are analogous to those of previous studies. One such study has obtained similar results, where propolis showed more efficiency in reducing the GI and PI than CHX [12]. Pereira et al. [39] evaluated efficacy of 5% Brazilian Green Propolis on gingivitis and found a statistically significant decrease in the average GI score and the plaque accumulation after 45 and 90 days of use of mouth wash when compared to baseline data. They concluded that alcohol-free 5% Brazilian green propolis mouthwash is efficacious for plaque control as well as gingivitis. Another study studied the effectiveness of 3% ethanolic propolis in reducing gingivitis caused by dental plaque [40]. Recent studies have revealed the natural therapeutic advantages possessed by the propolis, which can be believed to be a potential non-pharmacological consideration for the treatment of gingivitis and periodontitis (CP) [41].

A randomized, double-blind, placebo-controlled clinical trial [42] noted a reduction in papillary bleeding score (PBS), an indicator of gingival inflammation, that was similar in both 0.12% chlorhexidine and 2% alcohol-free typified propolis mouth rinse after 28 days. Their sub-group analysis of patients under the age of 40 revealed a statistically significant difference between the mean PBS scores of the test product over the gold standard chlorhexidine. Bretz et al. [43] conducted a randomized, double-blind, co-twin controlled clinical trial in 2014, comparing 2% typified propolis against a colour-matched 0.05% sodium fluoride (NaF) with 0.05% cetylpyridinium chloride (CPC) rinse positive control, and concluded that PBS was equivalent between both groups after 21 days of induced gingivitis. A systematic review in 2020 [12] concluded in favour of the clinical efficacy of propolis mouth rinse for plaque control and gingivitis. In February 2021, López-Valverde et al. [44] published a systematic review with meta-analysis concluding that propolis delivered in different forms may be an alternative in treating periodontal diseases and during supportive periodontal therapy. These research findings could be attributed to the antibacterial properties of propolis and the ability of the propolis to form calcium phosphates on the tooth surface, which may play a role in preventing the formation of dental plaques [45].

A literature review depicts contradictory findings as well, and studies have shown that propolis as a mouth wash was not as effective as CHX in reducing plaque. However, they were better at reducing the gingival inflammation [46–48]. A meta-analysis of English and Chinese literature by Hwu in 2014 [49] concluded that propolis did not provide statistically significant (*p* = 0.06) reduction in dental plaque. The shorter duration of the studies and the varying follow-up duration could have affected the results, which is a factor to be considered while comparing the results of the studies [50]. The varying composition of the propolis preparation and the propolis of different regions itself could have influenced the results of the studies [48].

The major limitation of our study is the short duration of study. The recommended evaluation period for any anti-plaque and anti-gingivitis agent is 6 months as mentioned by Halboub [12] and Gansolley [51]. A larger sample size, comprising a varied sociodemographic background, would allow the results obtained to be generalizable. Typification of the propolis used can be conducted to identify the biologically active components in the future. It is always better to know the exact details of biological active components present in botanical standardization, as specified by some studies [43]. This can be performed by using high-performance liquid chromatography, as demonstrated by Pereira et al. prior to their phase II study [39]. The present study could not perform this due to the shortage of research funds. Blinding formulations of mouthwash in terms of colour and taste could potentially further reduce bias in future crossover studies. The different indices and parameters used between studies could be standardized in the future to allow for easily comparable results. A suitable concentration of propolis for maximum efficacy could be a possible point of interest.

## Conclusion

Based on the results of the present study, it can be concluded that propolis can play a potential role in the maintenance of oral health by helping in the maintenance of gingivitis and dental plaque. These results are comparable to the standard chlorhexidine mouthwash. However, the results of the present study indicate the need for the further long-term studies using larger sample size on the Malaysian propolis to further establish its role on oral health.

## Data Availability

The datasets used in the present study are available from the corresponding author on reasonable request.
